# Childhood-onset hypertrophic cardiomyopathy caused by thin-filament sarcomeric variants

**DOI:** 10.1136/jmg-2023-109684

**Published:** 2024-01-31

**Authors:** Gabrielle Norrish, Marisa Gasparini, Ella Field, Elena Cervi, Juan Pablo Kaski

**Affiliations:** 1 Centre for Paediatric Inherited and Rare Cardiovascular Disease, Institute of Cardiovascular Science, University College London, London, UK; 2 Centre for Inherited Cardiovascular Diseases, Great Ormond Street Hospital For Children NHS Foundation Trust, London, UK

**Keywords:** Cardiomyopathies, Pediatrics, Phenotype

## Abstract

Up to 20% of children with sarcomeric hypertrophic cardiomyopathy (HCM) have disease-causing variants in genes coding for thin-filament proteins. However, data on genotype-phenotype correlations for thin-filament disease are limited. This study describes the natural history and outcomes of children with thin-filament-associated HCM and compares it to thick-filament-associated disease.

Longitudinal data were collected from 40 children under 18 years with a disease-causing variant in a thin-filament protein from a single quaternary referral centre. Twenty-one (female n=6, 35.5%) were diagnosed with HCM at a median age of 13.0 years (IQR 8.3–14.0). Over a median follow-up of 5.0 years (IQR 4.0–8.5), three (14.3%) experienced one or more major adverse cardiac events (MACE) (two patients had an out-of-hospital arrest and eight appropriate implantable cardiac defibrillator (ICD) therapies in three patients). One gene carrier died suddenly at age 9 years. Compared with those with thick-filament disease, children with thin-filament variants more commonly experienced non-sustained ventricular tachycardia [NSVT; n=6 (28.6%) vs n=14 (10.8%), p=0.024] or underwent ICD insertion (thin, n=13 (61.9%) vs thick, n=50 (38.5%), p=0.040). However, there was no difference in the incidence of MACE (thin 2.47/100 pt years (95% CI 0.80 to 7.66) vs thick 3.63/100 pt years (95% CI 2.25 to 5.84)) or an arrhythmic event (thin 1.65/100 pt years (95% CI 0.41 to 6.58) vs thick 2.55/100 pt years (95% CI 1.45 to 4.48), p value 0.43).

This study suggests that adverse events in thin-filament disease are predominantly arrhythmic and may occur in the absence of hypertrophy, but overall short-term outcomes do not differ significantly from thick-filament disease.

Up to 15%–20% of children with sarcomeric hypertrophic cardiomyopathy (HCM) have disease-causing variants in genes coding for thin-filament proteins.^1^ Initial studies in adults with HCM have suggested that thin-filament disease may be associated with an increased risk of malignant ventricular arrhythmias in the absence of severe hypertrophy.^2 3^ However, subsequent studies have suggested a more variable phenotype, in keeping with that observed in sarcomeric HCM more broadly.^4^ In children, data on genotype-phenotype correlations for thin-filament disease are limited.^3 5^ This study describes the natural history and outcomes of children with thin-filament-associated HCM and compares it to previously reported cohorts of thick-filament-associated disease.^6 7^

Forty children (from 33 families) at our centre aged ≤18 years with a disease-causing variant in a thin-filament protein were included (online supplemental table 1). Variant pathogenicity was classified according to the American College of Medical Genetics (ACMG) guidelines.^8^ Of these, 21 (female n=6, 35.3%) were diagnosed with HCM at a median age of 13.0 years (IQR 8.3–14.0); 2 (10%) were diagnosed in infancy. Six (35.3%) were probands, 12 (70.6%) had a family history of HCM and 6 (37.5%) had a family history of sudden cardiac death (SCD). Nine patients (42.9%) were symptomatic at baseline (chest pain n=5, 23.8%; dyspnoea n=5, 23.8%; palpitations n=6, 28.6%; syncope/pre-syncope n=1, 4.8%; New York Heart Association (NYHA) >1 n=2, 9.5%). One patient, with an additional variant of unknown significance variant in ACTC1, had left ventricular outflow tract obstruction. Eight patients (38.0%) underwent cMRI within 1 year of baseline assessment, of whom six (75.0%) had late gadolinium enhancement (LGE). Thirteen (68.4%) of gene carriers had a cMRI, of whom none had LGE detected. Three patients (15.8%) had NSVT (defined as three or more consecutive ventricular beats at a rate of greater than 120 beats/min with a duration of less than 30 s on ambulatory ECG monitoring) detected on initial ambulatory ECG. Seven patients (33.3%) had ‘complex’ genotypes with an additional variant of interest identified in other genes previously associated with inherited heart muscle disease. Patients with an additional variant were more likely to be diagnosed in infancy (n=2 vs n=0, p=0.042) but did not otherwise differ (online supplemental table 2).

10.1136/jmg-2023-109684.supp1 Supplementary data

Patients were followed up for a median of 5.0 years (IQR 4.0–8.5 years). Median age at last review was 18.0 years (IQR 15.0–20.0). During follow-up, 6 patients (33.3%) had NSVT detected on ECG monitoring, 13 (61.9%) underwent ICD implantation (11 (84.6%) for primary prevention) and 1 (4.8%) underwent surgical myectomy at age 2 years.

Three patients (14.3%) experienced one or more major cardiac events (MACE), all of which were arrhythmic; one patient presented with out-of-hospital arrest (OOHA) and had an ICD inserted for secondary prevention with one subsequent appropriate ICD therapy; one patient experienced an OOHA and had an ICD inserted for secondary prevention with three subsequent appropriate ICD therapies; and one patient with a primary prevention ICD had four appropriate ICD therapies over follow-up. No patients with a phenotype of HCM died or underwent cardiac transplantation but one gene carrier (predictively tested for *TNNT2* variant) without LVH and a normal 12 lead ECG died suddenly at age 9 years. This patient had not had cMRI previously. No differences were identified in the clinical phenotype between those who experienced a MACE and those that did not.

Table 1compares this cohort with previously published thick filament-related HCM (*MYH7* n=68, *MYBPC3* n=62).^6 7^ Patients with thin-filament disease were older at last follow-up and had smaller left atrial Z scores. There was no difference in the incidence of MACE (thin filament 2.47/100 pt years (95% CI 0.80 to 7.66) vs thick filament 3.63/100 pt years (95% CI 2.25 to 5.84)) or an arrhythmic event (thin filament 1.65/100 pt years (95% CI 0.41 to 6.58) vs thick filament 2.55/100 pt years (95% CI 1.45 to 4.48), p value 0.43). However, children with thin-filament variants more commonly experienced NSVT (n=6 (28.6%) vs n=14 (10.8%), p=0.024) and underwent ICD insertion (n=13 (61.9%) vs n=50 (38.5%), p=0.040) during follow-up.

**Table 1:** Comparison of characteristics of patients with thin and thick filament variants

	Thin filament (n=21)	Thick filament (n=130)^6 7^	P value
Age at diagnosis (median)	13.0 (IQR 8.3–14.0)	10.6 (IQR 5.0–14.1)	0.361
Symptoms at baseline	9 (43.9%)	51 (39.2%)	0.753
Medication at baseline	10 (47.6%)	48 (36.9%)	0.350
Baseline echocardiogram			
LVOT gradient ≥30 mm Hg	1 (4.8%)	18 (13.8%)	0.244
Median LVOT gradient (mm Hg)	6.0 (IQR 5.0–10.0)	8.0 (IQR 5.0–14.5)	0.257
MLVWT (median, IQR) at baseline	15.0 (IQR 9.5–25.5)	15.5 (IQR 11.0–23.)	0.864
MLVWT Z-score (median, IQR) at baseline	7.6 (IQR 1.5–16.7)	9.6 (IQR 5.3–15.9)	0.227
LA diameter, mm (mean±SD) at baseline	28.7±9.3	32.1±8.0	0.390
LA Z-score (mean±SD) at baseline	−0.3±3.0	1.6±2.1	0.035
Outcomes			
Median age at last follow-up	18.0 (IQR 15.0–20.0)	15.8 (IQR 11.4–17.4)	0.009
ICD insertion	13 (61.9%)	50 (38.5%)	0.043
Myectomy	1 (4.8%)	9 (6.9%)	0.712
OOHCA	2 (9.5%)	8 (6.2%)	0.564
NSVT on ambulatory ECG	6 (28.6%)	14 (10.8%)	0.026
MACE	3 (14.3%)	18 (13.8%)	0.957
Death	0	8 (6.2%)	0.243
Cardiac transplantation	0	5 (3.8%)	0.361

ICD, implantable cardiac defibrillator; LA, left atrium; LVOT, left ventricular outflow tract; MACE, major adverse cardiac events; MLVWT, maximal left ventricular wall thickness; NSVT, non-sustained ventricular tachycardia; OOHCA, out-of-hospital arrest .

In this single-centre cohort of children with HCM caused by thin-filament sarcomeric variants, there was a relatively high burden of symptoms, prevalence of NSVT on ambulatory monitoring and ICD implantation during follow-up. Although no patients with a phenotype of HCM died, three patients (almost one in six) experienced recurrent malignant ventricular arrhythmias and one gene carrier (*TNNT2*) with no evidence of LV hypertrophy died suddenly. Death occurring in gene carriers with no echocardiographic evidence of HCM has been reported by other studies,^4^ but due to the low frequency of such events, it is difficult to determine their risk. Future studies to investigate the arrhythmic burden of thin-filament gene carriers would be useful. Interestingly, no patients experienced any other adverse outcomes (death due to heart failure or cardiac transplantation) and only one required a myectomy—outcomes that are more frequently reported in larger unselected childhood cohorts.^9 10^ Arrhythmic events are the most common cause of mortality during childhood, but heart failure and arrhythmic events have been described to be more common from the second decade onwards.^10^ Longer term follow-up studies of those with thin-filament-associated disease would be helpful to determine the natural history of this patient group. Children with thin-filament variants more commonly underwent ICD insertion when compared with children with thick-filament variants, which is likely due to perceived high risk for malignant arrhythmias secondary to higher rates of NSVT detected during follow-up but could also be partly explained by an older age at last follow-up in the thin-filament group. NSVT is recognised to be a risk factor for sudden death in childhood HCM^11^ but the incidence of arrhythmic events or MACE in this study did not differ significantly between the groups. These data could suggest that the prognostic significance of NSVT could vary by genotype but our ability to detect a difference may also have been limited by small sample size as evidenced by the wide reported CIs representing uncertainty in the estimates. Our data suggest that adverse events over short-term follow-up in thin-filament disease are predominantly arrhythmic and could potentially occur in the absence of hypertrophy, but overall do not differ significantly from thick-filament disease.

## References

[1] Morita H , Rehm HL , Menesses A , et al . Shared genetic causes of cardiac hypertrophy in children and adults. N Engl J Med 2008;358:1899–908. 10.1056/NEJMoa075463 18403758 PMC2752150

[2] Moolman JC , Corfield VA , Posen B , et al . Sudden death due to troponin T mutations. J Am Coll Cardiol 1997;29:549–55. 10.1016/s0735-1097(96)00530-x 9060892

[3] Coppini R , Ho CY , Ashley E , et al . Clinical phenotype and outcome of hypertrophic cardiomyopathy associated with thin-filament gene mutations. J Am Coll Cardiol 2014;64:2589–600. 10.1016/j.jacc.2014.09.059 25524337 PMC4270453

[4] Pasquale F , Syrris P , Kaski JP , et al . Long-term outcomes in hypertrophic cardiomyopathy caused by mutations in the cardiac troponin T gene. Circ Cardiovasc Genet 2012;5:10–7. 10.1161/CIRCGENETICS.111.959973 22144547

[5] Maurizi N , Passantino S , Spaziani G , et al . Long-term outcomes of pediatric-onset hypertrophic cardiomyopathy and age-specific risk factors for lethal arrhythmic events. JAMA Cardiol 2018;3:520–5. 10.1001/jamacardio.2018.0789 29710196 PMC6128509

[6] Field E , Norrish G , Acquaah V , et al . Cardiac myosin binding protein-C variants in Paediatric-onset hypertrophic cardiomyopathy: natural history and clinical outcomes. J Med Genet 2022;59:768–75. 10.1136/jmedgenet-2021-107774 34400558 PMC7613139

[7] Norrish G , Kadirrajah V , Field E , et al . Childhood hypertrophic cardiomyopathy caused by beta-myosin heavy chain variants is associated with a more obstructive but less arrhythmogenic phenotype than myosin-binding protein C disease. Circ Genom Precis Med 2023;16:483–5. 10.1161/CIRCGEN.123.004118 37387224

[8] Richards S , Aziz N , Bale S , et al . Standards and guidelines for the interpretation of sequence variants: a joint consensus recommendation of the American college of medical genetics and genomics and the association for molecular pathology. Genet Med 2015;17:405–24. 10.1038/gim.2015.30 25741868 PMC4544753

[9] Alexander PMA , Nugent AW , Daubeney PEF , et al . Long-term outcomes of hypertrophic cardiomyopathy diagnosed during childhood: results from a national population-based study. Circulation 2018;138:29–36. 10.1161/CIRCULATIONAHA.117.028895 29490994

[10] Marston NA , Han L , Olivotto I , et al . Clinical characteristics and outcomes in childhood-onset hypertrophic cardiomyopathy. Eur Heart J 2021;42:1988–96. 10.1093/eurheartj/ehab148 33769460 PMC8139852

[11] Norrish G , Cantarutti N , Pissaridou E , et al . Risk factors for sudden cardiac death in childhood hypertrophic cardiomyopathy: a systematic review and meta-analysis. Eur J Prev Cardiol 2017;24:1220–30. 10.1177/2047487317702519 28482693

