# Supplementary material for: Childhood-onset hypertrophic cardiomyopathy caused by thin-filament sarcomeric variants
Source: J Med Genet. 2024 Jan 31;61(5):420–2. doi: 10.1136/jmg-2023-109684 (PMC11041581; doi:10.1136/jmg-2023-109684)
Supplement: Supplementary data [file jmg-2023-109684supp001.pdf]

Gender	Gene	Amino acid change	Molecular consequence	ACMG classification	Additional mutation gene	Amino acid change	Molecular consequence	ACMG classification	Outcome
M	TNNT2	p.Arg104His	Missense	P	N/A				Resuscitated OOHCA, NSVT on Holter, ICD insertion for secondary prevention with appropriate ICD discharge
F	TNNT2	p.E173del, c.517_519delGAG	Deletion	P/LP	No				NSVT on Holter, ICD insertion for primary prevention
M	TNNT2	p.Arg92Trp	Missense	P	No				NSVT on Holter, ICD insertion for primary prevention
M	TNNT2	p.Lys282Glu	Missense	VUS*	No				ICD insertion for primary prevention (MWT 28mm, LGE on CMRI, 12% 5-year risk SCD on HCM Risk Kids model)

F	TNNT2	p.Arg94His	Missense	P	No				
Not known	TNNT2	p.Ile89Asn	Missense	P	No				ICD insertion for primary prevention
M	TNNT2	p.Arg278Cys	Missense	LP	MYH7	p.Asp382Tyr	missense	LP	NSVT on Holter
M	TNNT2	unavailable			MYH7	p.Ala355Thr	missense	P/LP	
F	TNNT2	p.Ala114Val	Missense	P	MYBPC3	p.Arg502Trp	missense	VUS	NSVT on Holter, ICD insertion for primary prevention (NSVT)
M	TNNT2	p.Arg285Cys	Missense	LP	MYBPC3	p.Arg495Gln	missense	P/LP	ICD insertion for primary prevention (MWT 35mm, LGE on CMRI)
F	TNNT2	p.Glu173del	Deletion	P	No				
F	TNNI3	p.Lys206Gln	Missense	P	DES	p.Leu470Phe	missense	VUS	Resuscitated OOHCA, ICD insertion for secondary prevention with appropriate ICD discharge
Not known	TNNI3	p.Ala157Val	Missense	P	No				
Not known	TNNI3	p.Arg162Gln	Missense	P	No				ICD insertion for primary prevention
M	TPM1	p.Glu192Lys	Missense	P/LP	No				ICD insertion for primary

									prevention (MWT 34mm, LGE on CMRI, NSVT on exercise testing)
M	TPM1	p.Glu192Lys	Missense	P/LP	MYH7	p.ALa100Thr	missense	VUS	NSVT on Holter, ICD insertion for primary prevention
M	TPM1	p.Tyr221Cys	Missense	VUS*	No				NSVT on Holter, ICD insertion for primary prevention (NSVT, MWT 30mm) with appropriate ICD discharge
M	TPM1	p.Asp175Asn	Missense	P	No				ICD insertion for primary prevention (MWT 22, NSVT, LGE on CMRI)
Not known	TPM1	p.Glu192Lys	Missense	P/LP	No				
M	ACTC	p.Glu101Lys	Missense	P	No				
F	ACTC	p.Arg97Ser	Not reported	VUS^	TNNC1	p.Asp145Glu	missense	VUS	

Supplementary table 1: Thin filament genetic variants

3 patients with a variant of unknown significance were included in the cohort after review of the clinical and genetic data confirmed there was sufficient evidence that the variants are likely disease-causing in these individuals. *included in the cohort as segregation in family members. ^ VUS in ACTC1 with mosaicism in myocardial tissue and additional VUS in TNNC1 gene

	**Simple genotype (n=14)***	**Complex genotype (n=7)**	**P**
Female	3/10 (30%)	3 (42%)	0.585
Diagnosed in infancy	0	2 (28.5%)	0.042
Family history HCM	7/10 (70%)	5 (71%)	0.949
Family history SCD	3/9 (33%)	3 (42%)	0.696
Proband	3/10 (30%)	3 (42%)	0.585
**Baseline**			
Any symptom	6/14 (42.9%)	3 (42.9%)	1.000
Atypical distribution	3/13 (23.1%)	3 (42%)	0.357
Any medication	4/14 (28.6%)	6 (85.7%)	0.013
NSVT on Holter	2/14 (16.7%)	1 (14.3%)	0.891
**Follow up**			
Any symptom	4/14 (28.6%)	2 (28.6%)	1.000
Any medication	7/14 (50%)	1 (14.3%)	0.112
NSVT on Holter	3/11 (27.2%)	3 (42.9%)	0.494
ICD implantation	9/14 (64.3%)	4 (57.1%)	0.751
Appropriate ICD therapy	2/14 (14.3%)	1 (14.3%)	1.000
Inappropriate ICD therapy	0	1 (14.3%)	0.147
Sustained ventricular arrhythmia	2/14 (14.3%)	1 (14.3%)	1.000

Supplementary table 2: Comparison of patients with simple and complex genotypes
*N= 14 unless otherwise indicated
